# Angiographic Characteristics of Cerebral Perfusion and Hemodynamics of the Bridging Artery After Surgical Treatment of Unilateral Moyamoya Disease

**DOI:** 10.3389/fnins.2022.922482

**Published:** 2022-06-14

**Authors:** Kun Zhang, Wei Ren, Yu-Xue Sun, Xin-Jun Wang, Chao-Yue Li, Zi-Liang Wang, Tian-Xiao Li, Bu-Lang Gao

**Affiliations:** ^1^The Fifth Affiliated Hospital of Zhengzhou University, Zhengzhou, China; ^2^Zhengzhou University People’s Hospital, Henan Provincial People’s Hospital, Zhengzhou, China

**Keywords:** moyamoya disease, digital subtraction angiography, computational fluid dynamics, bypass grafting, hemodynamic stresses

## Abstract

**Purpose:**

To investigate the characteristics of cerebral perfusion and hemodynamics of bypass grafting in the treatment of moyamoya disease (MMD) using the iFlow color-coded flow map in comparison with magnetic resonance imaging–perfusion-weighted imaging (MRI–PWI) and computational fluid dynamic (CFD) analysis.

**Materials and Methods:**

Patients with MMD treated with bypass grafting who had undergone MRI PWI and digital subtraction angiography for iFlow color-coded map was retrospectively enrolled and CFD was performed for calculating the hemodynamic stresses around the bypass grafting.

**Results:**

Forty-five patients with unilateral MMD treated with bypass surgery were enrolled. The bypass surgery was successful in all patients, with no severe neurological complications during the periprocedural period. Followed up for 4–12 months (median 5.5), the neurological function was good in all patients. The cerebral blood flow (CBF), cerebral blood volume (CBV), mean transit time (MTT), and time to peak (TTP) were significantly (*p* < 0.05) improved in the middle cerebral artery distribution area on the surgical side before and after vascular bypass, and the difference of TTP (s) measured from the proximal bifurcation of common carotid artery to the confluence of sinus was also significant (*p* < 0.05). A significant (*p* < 0.05) positive correlation existed in the perfusion parameters between the iFlow blood perfusion and the MRI–PWI perfusion, with *r*-value for TTP of 0.765 (*p* < 0.01). The iFlow color-coded blood flow map showed warm color changes on the diseased side, similar to those on the contralateral side. In CFD analysis, the hemodynamic stresses were all improved, in and around the bypass grafting and distal vessels, which were beneficial to blood flow entering distal arterial branches.

**Conclusion:**

The iFlow color-coded flow map can be used to analyze cerebral perfusion after bypass grafting for MMD, similar to MRI–PWI, and CFD can be used to analyze the hemodynamics after bypass grafting, revealing improved hemodynamics to promote blood flow entering distal arteries.

## Introduction

Moyamoya disease (MMD) is a chronic occlusive intracranial arterial disease of unknown etiology with the characteristics of steno–occlusive alterations at the terminal portions of the internal carotid artery and an abnormal vascular network at the base of the brain (Suzuki and Takaku, 1969; Kato et al., 2019; Chen et al., 2020; Lee et al., 2020). In a Japanese survey, patients with MMD generally have bilateral involvement while some patients also manifest as unilateral involvement (Kuriyama et al., 2008). For the patients with definitive causes of the steno–occlusive changes, moyamoya syndrome is used. Moyamoya disease may be an uncommon cause of non-atherosclerotic cerebrovascular disease, especially in East Asia, where isolated stenosis of the middle cerebral artery (MCA) is frequently observed in young patients and may ultimately evolve into MMD (Choi et al., 2007). Digital subtraction angiography (DSA) has become an important tool for assessing MMD before surgery because it can comprehensively evaluate the bridge vessel and the whole cerebral hemodynamics in patients with MMD.

The Syngo iFlow color-coded blood flow imaging software in the Siemens Artis angiographic system (Artis Zeego; Siemens Healthcare, Forchheim, Germany) synthesizes 2D DSA imaging data into a color image with rich blood perfusion information through different color codes; thus, enabling analyzing blood flow peak time, tissue perfusion, and other blood flow change parameters as well as obtaining the whole brain perfusion at the same time (Fang et al., 2021). The 3D imaging data obtained through rotational angiography of DSA can be used for computational fluid dynamics (CFD) analysis of hemodynamic stresses including total pressure and wall shear stress (Gao et al., 2013, 2022); thus, beneficial to a better understanding of disease evolution of MMD after surgery. Currently, no in-depth studies have been performed on CFD analysis of hemodynamics of the bridging vessels in MMD and the cerebral perfusion assessed by iFlow color-coded flow imaging software in the world. It was hypothesized that combination of the CFD hemodynamic analysis and Syngo iFlow analysis of cerebral perfusion would be sufficient to evaluate changes in hemodynamic stresses and cerebral perfusion of unilateral MMD after surgery. The purpose of this study was to assess the feasibility of using these two techniques in analyzing cerebral perfusion and hemodynamic characteristics of MMD after surgery.

## Materials and Methods

### Subjects

This retrospective cohort one-center study was performed between December 2019 and April 2021 in a tertiary hospital after the approval by the ethics committee of the hospital. Patients or their family members had signed the informed consent to participate. Unilateral or bilateral MMD was diagnosed by DSA or magnetic resonance imaging (MRI) according to relevant studies and the guideline for the management of MMD (Suzuki and Takaku, 1969; Research Committee on the Pathology, and Treatment of Spontaneous Occlusion of the Circle of Willis, and Health Labour Sciences Research Grant for Research on Measures for Infractable Diseases, 2012; Fujimura et al., 2022). Patients with unilateal MMD (stenosis or occlusion at the end of internal carotid artery or at the initial segment of anterior cerebral artery or middle cerebral artery), manifestation of ischemic cerebral symptoms, abnormal vascular network, treatment of bypass surgery, and DSA examinations before and after surgery were enrolled. The surgical indications were by the patients with unilateral MMD who manifested as ischemic cerebral symptoms (Fujimura et al., 2022), and the surgical treatment scheme was a combination of brain–dura mater–muscle vascular application and superficial temporal artery (STA)–MCA anastomosis. Exclusion criteria were by the patients with moyamoya syndrome caused by certain diseases like immune diseases, sickle cell anemia, and history of intracranial radiotherapy. Patients with potential cardiac emboli such as atrial fibrillation, infective endocarditis, recent myocardial infarction, or dilated cardiomyopathy were also excluded.

Preoperative comprehensive cerebral angiography combined with magnetic resonance imaging perfusion-weighted imaging (MRI PWI) of cerebral blood flow (CBF) was conducted to determine the bypass area. Follow-up DSA was performed at 4–12 (5.5 ± 4.3) months after surgery, and 1–2 days before DSA follow-up angiography, MRI–PWI was performed to evaluate cerebral perfusion. The changes of the bypass hemodynamics and CBF perfusion were evaluated at the same time. The modified Rankin Scale score (mRS) was used to assess the neurological status of the patients.

### Magnetic Resonance Imaging Perfusion-Weighted Imaging

Magnetic resonance imaging was conducted with a 3-T MR scanner (Magnetom, Siemens, Erlangen, Germany). Conventional MRI of the head was performed in the axial and coronal projections using the following parameters: T2-weighted turbo spin echo (TSE) with fat saturation, T1-weighted TSE, and postcontrast fat-saturated T1-weighted TSE. Perfusion-weighted imaging was acquired with a 3D T1-weighted spoiled gradient-echo sequence using the following parameters: TR/TE53.5/1.13 ms, 230 mm × 230 mm for the field of view, and 108 × 128 matrix.

### Digital Subtraction Angiography and Imaging Data Collection

The Siemens Artis angiographic system were used for the DSA examination. After femoral artery access, the DSA cerebral angiography was performed for observing the bypass blood flow and cerebral perfusion. After the distal end of a 4F contrast catheter was placed 4 cm below the carotid artery bifurcation, cerebral angiography was performed with the contrast agent of iodixanol (100 ml/piece, Jiangsu Hengrui medicine, China), contrast agent flow rate of 5 ml/s, total injection dose of 10 ml, injection delay of 1 s, high-pressure syringe pressure of 300 PSI, and frame number of 4 fp/s until the venous sinus phase.

### Syngo iFlow Analysis

The imaging data were sent to the workstation for Syngo iFlow analysis. A complete sequence of 2D-DSA images was synthesized into a cold and warm color map. Blue indicated the longest delay time after contrast agent injection, and red indicated the shortest delay time after contrast agent injection. Time to peak (TTP) was defined as the time when the density of the region of interest/point reached the peak at the condition when the parameters such as the total amount and rate of contrast medium were fixed. Through point measurement, the time density curve at the point of interest was obtained. The rising curve represented the contrast medium filling stage and the falling curve represented the contrast medium emptying stage. The changes of TTP after vascular bypass grafting and on the healthy side directly and accurately reflected the differences and changes in the blood flow between the operative side and the normal healthy side. The contrast agent TTP at the point of interest was recorded at the superior sagittal sinus, the anastomosis of bypass, and the bifurcation point of M1 segment of the MCA.

### Computational Fluid Dynamic Modeling and Hemodynamic Analysis

Three-dimensional DSA imaging data in DICOM were used to establish the CFD model for hemodynamic analysis. The Amira software (version 5.2.2 Visual Computing Lab, ISTI, CNR) was used to reconstruct the imaging data for surface rendering, and the Meshlab software (Meshlab version 1.3.3, Visual Computing Lab, ISTI, CNR) was applied for surface smoothing and reconstruction of arteries. Finite-volume solution was conducted with the fluent software (version 12.0.16, Ansys, Lebanon, NH, United States), with blood viscosity coefficient μ and blood density ρ being set at 0.00345 Pa and 1050 kg/m^3^, respectively. The inlet speed was set at 0.32 m/s according to the measured average inlet speed of healthy adult volunteers, and the pressure at the outlet was set at 0 Pa. Post-processing was performed with the EnSight software (version 9.0; CEI, Apex, NC, United States) for the analysis of the hemodynamic stresses including the total pressure and wall shear stress.

### Statistical Analysis

The statistical analysis was performed with the SPSS 19.0 (IBM, Chicago, IL, United States). Measurement data in normal distribution were presented as mean ± standard deviation and tested with the paired *t*-test. If in skew distribution, the measurement data were presented as median and interquartile range and tested with the Mann–Whitney *U* test for comparisons between the groups. Enumeration data were presented as frequency and percentages and tested with the Chi-squared test. Pearson correlation was performed between different parameters; values are considered significant when *p* < 0.05.

## Results

**FIGURE 1:**
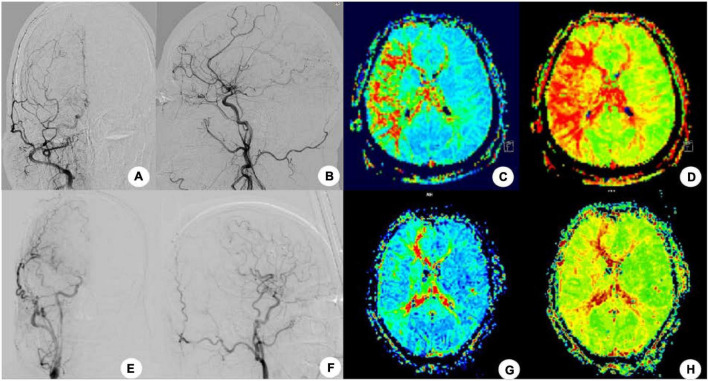
After right superficial temporal artery-middle cerebral artery (STA-MCA) bypass grafting in one patient, no recurrence of stroke and transient ischemic attack was found at 4-month follow-up, and cerebral angiography showed that the bypass supplied blood to the brain. **(A,B)** Cerebral angiography before the bypass surgery was shown. **(C,D)** Mean transit time **(C)** and TTP **(D)** were shown before surgery in MRI-PWI. **(E,F)** Cerebral angiography after surgery. **(G,H)** Four months after surgery, the MTT **(G)** and TTP **(H)** were significantly improved.

**FIGURE 2:**
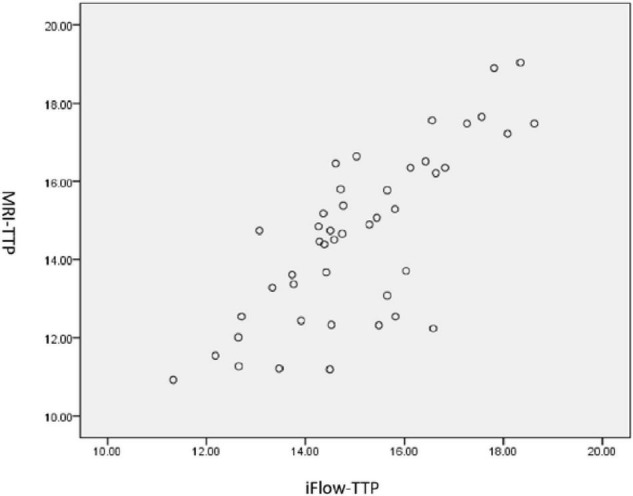
Pearson correlation in TTP between MRIPWI and iFlow outcome in patients with moyamoya.

During the period of study, 362 patients with bilateral MMD underwent surgical treatment, including 63 patients with unilateral MMD. Among 63 patients with unilateral MMD, surgery was successfully performed in all patients with the success rate of 100%. Among the 55 patients with unilateral MMD who were followed up angiographically, 45 patients with unilateral MMD who had been confirmed to have patent bypass arteries after surgery were enrolled, including 20 male and 25 female patients aged 51.0 ± 4.1 (range, 21–62) years. The first clinical manifestation was ischemia in 29 cases and hemorrhage in 16 cases. At admission to the hospital, the mRS was 3 in 4 patients, 2 in 26, 1 in 10, and 0 in 5. The bypass surgery was successful in all patients, with no severe neurological complications during the periprocedural period except for reversible neurological dysfunction in two (4.44%) patients and small cerebral infarction in one (2.22%). Followed up for 4–12 months (median 5.5) after the bypass grafting surgery, except for two patients who had ischemic strokes, no patients had ischemic strokes. The neurological function was good in all patients, with the mRS score less than or 2. Vascular bypass grafting had resulted in significant improvement in the neurological status. Digital subtraction angiography follow-up showed patent bypass grafting, with blood flow entering from the STA into intracranial regions (Figure 1), with significantly improved MTT and TTP compared with those before surgery. The CBF, cerebral blood volume (CBV), mean transit time (MTT), and TTP were significantly (*p* < 0.05) improved in the MCA distribution area on the surgical side before and after vascular bypass in patients with MMD, and the difference of TTP (s) measured from the proximal bifurcation of common carotid artery to the confluence of sinus was also significant (*p* < 0.05) (Table 1). A significant (*p* < 0.05) positive correlation existed in the perfusion parameters between the iFlow blood perfusion and the MRI–PWI perfusion, with the *r*-value for TTP of 0.765 (*p* < 0.01) (Figure 2).

**TABLE 1:** The MRI–PWI perfusion parameters in MCA distribution area before and after vascular bypass grafting.

Variables	Pre-surgery	Post-surgery	Statistical value	*p*
CBF (ml⋅100 g^–1^⋅min^–1^)	38 (32.43–43.50)	96.4 (77.50–101.51)	–4.35	0.000
CBV (ml⋅100 g^–1^)	4.28 ± 2.04	5.11 ± 2.51	–1.28	0.194
MTT	5.85 ± 2.16	4.06 ± 2.05	3.01	0.005
TTP	18.59 ± 3.07	13.91 ± 3.65	4.91	0.000
TTP difference	14.40 ± 2.32	8.40 ± 3.75	6.80	0.000

*MRI, magnetic resonance imaging; PWI, perfusion-weighted imaging; MCA, middle cerebral artery; CBF, cerebral blood flow; CBV, cerebral blood volume; MTT, mean transit time; TTP, time to peak; TTP difference, between proximal bifurcation of common carotid artery to sinus confluence.*

**FIGURE 3:**
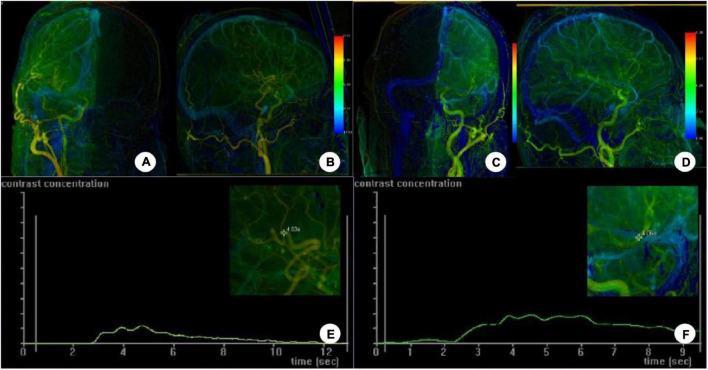
The iFlow color-coded blood flow map was constructed on DSA postoperative cerebral angiographic images. **(A–D)** Angiography through the external carotid artery showed warm color changes on the diseased side **(A,B)**, similar to those on the contralateral side **(C,D)**. **(E,F)** The time–density curve at the anastomosis and region of interest around the upper and lower trunk of the MCA showed that the contrast agent apparently disappeared at the anastomosis while the contrast agent peak value was significantly increased at the upper and lower trunk of the MCA. The interval from the upper or lower MCA trunk to the upper middle sagittal sinus was roughly the same.

The iFlow color-coded blood flow map from postoperative DSA cerebral angiographic images showed warm color changes on the diseased side (Figure 3), similar to those on the contralateral side. The time–density curve at the anastomosis and region of interest around the upper and lower trunk of the MCA showed that the contrast agent apparently disappeared at the anastomosis while the contrast agent peak value was significantly increased at the upper and lower trunk of the MCA. The interval from the upper or lower MCA trunk to the upper middle sagittal sinus was roughly the same.

**FIGURE 4:**
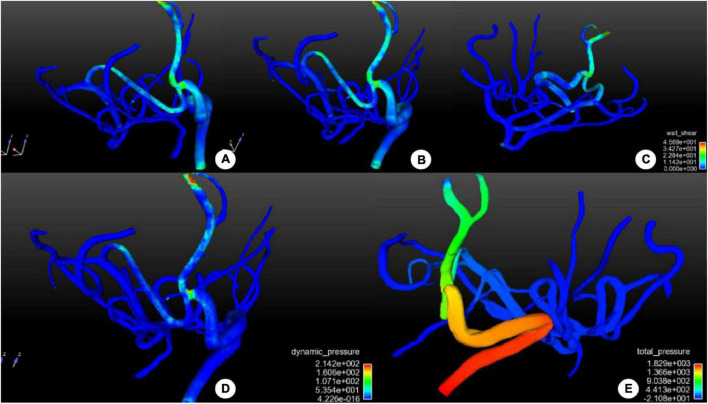
Distribution of hemodynamic stresses in and around the surgical bypass vessel after bypass surgery. **(A–C)** Distribution of wall shear stress (WSS) in and around the bypass vessel was shown. No abnormal increase was demonstrated in WSS in the bypass or the anastomosis, and the middle cerebral artery supply blood reversely without producing abnormal WSS. **(D)** The distribution of dynamic pressure was shown, with increased dynamic pressure around the anastomosis to promote blood flow distally, but the dynamic pressure was stable in the whole vessels. **(E)** The distribution of total pressure was shown in and around the bypass vessel. Proximal to the anastomosis, the total pressure was high to promote blood flow distally. A low total pressure was shown in the distal branches of the middle cerebral artery, beneficial to blood entering distal branches. The color scale represents the size of the value.

Computational fluid dynamic analysis was performed for analyzing the hemodynamic stresses in and around the surgical bypass vessel (Figure 4). The wall shear stress was improved in the bypass or anastomosis, suggesting high-speed blood flow entering the distal arterial branches. The distribution of dynamic pressure was slightly increased around the anastomosis, but stable dynamic pressure was present in the whole vessels. A higher total pressure was shown on the bypass grafting while a low total pressure was shown in the branches of the MCA distal to the anastomosis, which promoted blood flow to easily enter the distal vessels.

## Discussion

### Major Findings

This study investigated the characteristics of cerebral perfusion and hemodynamics of bypass grafting in the treatment of MMD using the iFlow color-coded flow map in comparison with MRI–PWI and CFD analysis. It was found that iFlow color-coded flow map could be used to analyze cerebral perfusion after bypass grafting for MMD, similar to MRI–PWI, and CFD could be used to analyze the hemodynamics after bypass grafting, revealing improved hemodynamics to promote blood flow entering distal arteries.

### Surgical Revascularization

Surgical revascularization for the patients with MMD along with cerebral ischemic attacks is able to decrease the risk of cerebral infarction and frequency of transient ischemic attack besides improving postoperative activity of daily life and long-term prognosis of neurocognitive function (Uchino et al., 2019; Ong et al., 2020; Kanoke et al., 2021; Wali et al., 2021; Fujimura et al., 2022). Surgical treatment is the first choice for MMD, including direct, indirect, and combined bypass. As the most popular procedure, direct bypass involves STA to MCA (STA–MCA) anastomosis (Hishikawa et al., 2016). STA–anterior cerebral artery and occipital artery–posterior cerebral artery bypass procedures have been used for the territory of anterior cerebral artery and posterior cerebral artery, respectively (Hayashi et al., 2009; Kawashima et al., 2010). Many indirect bypass procedures have been reported using various types of tissues as blood supply sources (Hishikawa et al., 2016); however, the direct bypass surgery should be the first-line approach for adult patients with MMD. For adult patients with MMD, an indirect revascularization surgery alone is not sufficient, and a surgery combining both indirect and direct revascularization is necessary based on the 2021 Japanese Guidelines for the management of patients with MMD (Fujimura et al., 2022) and multiple studies (Mizoi et al., 1996; Czabanka et al., 2009; Guzman et al., 2009; Kim et al., 2010; Lee et al., 2012; Cho et al., 2014; Deng et al., 2018; Jeon et al., 2018). A combined revascularization procedure can supply a wider territory of the ischemic brain than the direct procedure alone, leading to a better surgical outcomes for adult patients. After the bypass surgery, the blood is redistributed, and the blood flow in the bypass can enter the arterial branches distal to the anastomosis in the ischemic brain area, achieving the therapeutic effect. However, approximately 21.5–50% of patients who have undergone direct revascularization of MMD may experience cerebral hyperperfusion syndrome, especially on the third day after surgery (Lee et al., 2018), which is probably caused by transient dysfunction of cerebral autoregulation. Moyamoya disease is characterized by thin and atrophic intracranial arteries as well as formation of moyamoya arteries caused by long-term ischemia and metabolic insufficiency of blood perfusion. Surgical revascularization will lead to relatively excessive amount of increased blood perfusion in the chronically ischemic cerebral area and provoke various hyperperfusion symptoms, including headaches, intracerebral hemorrhage, and neurological deficits. These symptoms will be resolved following restoration of autoregulation of arteries and normalization of blood perfusion (Kim et al., 2008; Zhao et al., 2013).

### Value of Computational Fluid Dynamic in Evaluating Hemodynamic Stresses After Revascularization of Moyamoya Disease

Computational fluid dynamic analysis can be used to directly investigate the changes in the hemodynamic stresses in the bypass arteries and those distal to the anastomosis. Vascular anastomosis from extracranial to intracranial arteries can quickly improve the state of intracranial ischemia. With the STA which has good expansibility (Yanagihara et al., 2018), the treatment effect is relatively good with significant benefit. Bypass surgery changes the relative balance of fluid stress in intracranial arteries, and the hemodynamic stresses can be demonstrated by CFD. In CFD analysis, the overall vascular bed structure of the STA, bypass vessels and anastomosed MCA vascular network can be displayed clearly. Analyzing the vascular hemodynamics after bypass grafting is helpful to understand and evaluate the prognosis of patients with MDD. After the bypass surgery, blood enters the remaining vascular network of MCA and increases the flow of the vascular network, but it may also retrogradely hinder or reverse the compensatory blood flow from the anterior and posterior cerebral arteries. Many factors need to be considered when anastomosing the extracranial artery. The choice of anastomotic site should be balanced against the ischemic area, vascular network of the receptor, and even pressure difference after anastomosis. Our study showed that after bypass grafting, the vascular network distal to the anastomosis had low and stable total pressure and wall shear stress, which is beneficial to blood flow entering the network. The high total pressure of blood flow at the STA can promote blood flow into the MCA network, beneficial to the opening and expansion of the network. However, hyperperfusion syndrome should be prevented using medications early after revascularization.

The DSA high-resolution imaging can be used to obtain isotropic 3D volume data for arterial morphology measurement, hemodynamic simulation, and blood flow stress measurement (Gao et al., 2013, 2022). In this study, based on the 3D image data of MMD after bypass grafting, CFD technology was used to investigate hemodynamic stress changes, and combined perfusion was used to evaluate the significance of bypass grafting from a new perspective.

### Value of iFlow Color-Coded Blood Flow Map in Evaluation of Cerebral Perfusion

The iFlow color-coding technology can convert 2D-DSA image into parameter image of single component according to the emptying time of contrast agent. The difference of color represents the difference of peak filling rate of contrast agent in different regions of interest, and the difference of color temperature represents the maximal enhancement values of contrast agent. This is the color-coded image. Digital subtraction angiography is still the gold standard for evaluating the condition of MMD. By comparing the DSA images before and after bypass surgery in combination with the iFlow color-coding technology, quantitative changes of hemodynamics and cerebral perfusion can be investigated. Compared with other perfusion imaging techniques, iFlow color-coded blood flow map has the advantages of fast imaging and quantitative research, which makes up for conventional DSA which can only provide vascular architecture related information without perfusion quantitative information (Cattaneo et al., 2017). In this study, both iFlow flow map and MRI–PWI can quantitatively evaluate the perfusion of cerebral ischemic area in patients with MMD, and Pearson correlation analysis confirmed a high positive correlation in TTP of relative perfusion parameters obtained between the two imaging methods. Thus, DSA imaging plus iFlow color-coded map can be used to evaluate the quantitative changes in the hemodynamics of bypass vessel and cerebral perfusion, avoiding the use of additional medical resources like MRI–PWI for evaluation.

Bypass surgery is an effective measure to prevent stroke. In patients with MMD with unobstructed bypass vessels, the TTP time near the bifurcation of the MCA and the anastomosis of the bypass vessels to the middle of the sagittal sinus was approximately the same, which indicated no significant difference in cerebral perfusion between the ischemic side and the contralateral normal side. This explains the significance of the bypass vessel to the blood supply area of the MCA. The iFlow color-coded blood flow map showed that the time–density curve was different at the bypass vessel anastomosis from that near the bifurcation of the contralateral MCA. The time–density curve (TDC) peak was low, the waveform was gentle and the slope was low. From the TDC diagram, it can be seen that the TDC peak of the bypass vessel decreases rapidly by nearly 0, indicating that the vascular network of the MCA presents a “siphon effect” after the bypass vessel. The use of the iFlow color-coded flow map makes simultaneous DSA evaluation of whole brain perfusion examination a reality, without increasing the dosage of contrast agent. However, the imaging quality of syngo iFlow software is easily disturbed by moving artifacts, which requires the consistency of contrast acquisition conditions. In this study, the relative cycle time difference is used to compare the bilateral perfusion so as to decrease the disturbance.

In the past, evaluation of the effect of bypass surgery only relied on evaluating changes of patients’ symptoms and signs and image perfusion [such as computed tomography perfusion (CTP) and PWI for cerebral perfusion evaluation]. However, it is not sufficient because the assessment of the symptoms and signs has strong subjectivity and imaging perfusion evaluation needs additional examination. The examination of cerebral hemodynamics is intuitive, significant, and quantifiable, and early postoperative changes can reflect the changes in hemodynamics, with special advantages in evaluating the curative effect of bypass surgery. Cerebral perfusion can be performed with computed tomography perfusion and MRI–PWI. The iFlow color-coded flow map selects the enhancement peak time of TDC to determine the time node of DSA image reconstruction. From the outcome of our study on CFD hemodynamics and iFlow technique of flow map, it was found that the use of iFlow map based on DSA imaging datasets can be used to better evaluate the blood flow changes.

The change of CBV was inconsistent for different segments of MMD. Patients with mild ischemia have a compensatory increase in CBV before surgery, which will gradually become normal after surgery. Cerebral blood volume in patients with severe ischemia decreases before surgery but will gradually increase after surgery (Yin et al., 2018). However, the mean value of the CBV in this study slightly increased after surgery compared with that before surgery, which probably indicates that these patients had severe ischemia even though no significant difference was detected.

The advantage of color-coded flow map lies in the following: Fast imaging, quantitative and comprehensive evaluation of intraoperative CBF, clear display of changes of blood flow velocity and cerebral hemodynamics, and reflection of perfusion information of brain tissue. Compared with conventional MRI–PWI, it is easier to evaluate the perfusion changes before and after surgery. Computed tomography perfusion and MRI–PWI are effective and readily accessible to assess the cerebral perfusion status, which had been proved in several studies (Schubert et al., 2009; Saposnik and Strbian, 2018). The iFlow color-coded flow map is not better than CTP or PWI. Rather, iFlow color-coded flow map makes it possible to use the same DSA data to evaluate whole brain perfusion at the same time of DSA angiography without necessitating repeated examination at the cost of increasing the dosage of contrast agent. Fast and quantitative imaging of the iFlow color-coded flow map makes up for the fact that conventional DSA can only provide vascular architecture related information without perfusion quantitative information.

### Limitations

Some limitations existed in this study, including the retrospective and one-center design, enrollment of Chinese patients only, a small cohort of patients, and non-randomization, which may all affect the generalization of the outcomes. Moreover, the value of color-coded flow map was not clear for bilateral MMD, and further studies on patients with bilateral MMD is needed. Future studies will have to resolve all the issues for better outcomes.

## Conclusion

In conclusion, iFlow color-coded flow map can be used to evaluate blood flow perfusion in MMD after bypass grafting, similar to MRI–PWI, and CFD can be used to analyze the hemodynamics after bypass grafting, revealing improved hemodynamics to promote blood flow entering distal arteries.

## Data Availability Statement

The original contributions presented in this study are included in the article/supplementary material, further inquiries can be directed to the corresponding authors.

## Ethics Statement

The studies involving human participants were reviewed and approved by Ethics Committee of Henan Provincial People’s Hospital. The patients/participants provided their written informed consent to participate in this study. Written informed consent was obtained from the individual(s), and minor(s)’ legal guardian/next of kin, for the publication of any potentially identifiable images or data included in this article.

## Author Contributions

KZ and B-LG: study design. KZ, WR, Y-XS, C-YL, Z-LW, and T-XL: data collection. KZ, X-JW, and B-LG: data analysis. Z-LW: supervision. B-LG: revision. All authors approved the submitted version.

## Conflict of Interest

The authors declare that the research was conducted in the absence of any commercial or financial relationships that could be construed as a potential conflict of interest.

## Publisher’s Note

All claims expressed in this article are solely those of the authors and do not necessarily represent those of their affiliated organizations, or those of the publisher, the editors and the reviewers. Any product that may be evaluated in this article, or claim that may be made by its manufacturer, is not guaranteed or endorsed by the publisher.
